# Diverticular perforation of terminal ileum associated with chemotherapy for non-small cell lung carcinoma: a case report

**DOI:** 10.1093/jscr/rjad179

**Published:** 2023-04-12

**Authors:** So Kasuga, Shinya Abe, Hiroaki Nozawa, Kazuhito Sasaki, Koji Murono, Shigenobu Emoto, Hiroyuki Matsuzaki, Yuichiro Yokoyama, Yuzo Nagai, Yuichiro Yoshioka, Takahide Shinagawa, Hirofumi Sonoda, Tetsuo Ushiku, Soichiro Ishihara

**Affiliations:** Department of Surgical Oncology, The University of Tokyo, Tokyo, Japan; Department of Surgical Oncology, The University of Tokyo, Tokyo, Japan; Department of Surgical Oncology, The University of Tokyo, Tokyo, Japan; Department of Surgical Oncology, The University of Tokyo, Tokyo, Japan; Department of Surgical Oncology, The University of Tokyo, Tokyo, Japan; Department of Surgical Oncology, The University of Tokyo, Tokyo, Japan; Department of Surgical Oncology, The University of Tokyo, Tokyo, Japan; Department of Surgical Oncology, The University of Tokyo, Tokyo, Japan; Department of Surgical Oncology, The University of Tokyo, Tokyo, Japan; Department of Surgical Oncology, The University of Tokyo, Tokyo, Japan; Department of Surgical Oncology, The University of Tokyo, Tokyo, Japan; Department of Surgical Oncology, The University of Tokyo, Tokyo, Japan; Department of Pathology, The University of Tokyo, Tokyo, Japan; Department of Surgical Oncology, The University of Tokyo, Tokyo, Japan

**Keywords:** diverticulitis, ileum, perforation, chemotherapy

## Abstract

A 71-year-old man was diagnosed with advanced non-small cell lung carcinoma and treated with chemotherapy developed ileocecal diverticulitis three times over the last 2 months of receiving second-line treatment. During the fourth diverticulitis event, the patient presented with fever and abdominal pain, worsening after 5 days. Abdominal computed tomography showed ascites and intra-abdominal free air, suggesting bowel perforation with acute diffuse peritonitis. We performed emergency surgery; the surgical findings showed diverticulosis with perforated diverticula in the ileocecal region. We performed ileocecal resection, an ileostomy and a mucous fistula of the ascending colon. Histopathological examinations revealed pseudodiverticula at the perforation, where the mucosa was depressed through the muscularis propria. Hence, we diagnosed perforated ileal diverticulitis. Repeated diverticulitis triggered by chemotherapy might have resulted in perforation. Small bowel diverticula are rare, but diverticulitis can occur in patients receiving chemotherapy and with cases of unexplained fever and abdominal pain.

## INTRODUCTION

Colon diverticulosis is common, with a prevalence of ~42–60% [1–3], whereas small intestine diverticulosis, excluding Meckel’s diverticula, is rare, affecting 0.02–7%. Ileal diverticulosis is less common than jejunum diverticulosis. Small intestine diverticulosis is often asymptomatic and detected only during imaging or autopsy [1, 4].

This case reports a small intestinal diverticular perforation in a patient receiving chemotherapy for non-small cell lung cancer. Immunocompromised patients taking methotrexate or steroids or receiving chemotherapy are at a higher risk of developing severe diverticulitis that may not respond to conservative treatment [6–8]. Chemotherapy patients also face significantly higher perforation risk [8]. This is reportedly the first case of ileal diverticulitis perforation in a chemotherapy patient.

## CASE REPORT

**Figure 1:**
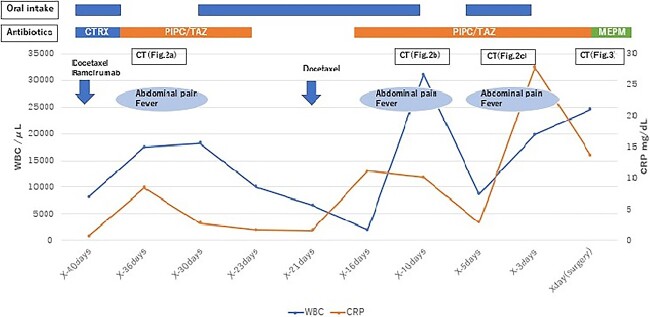
Flowchart showing the trend of WBC count and CRP level from second-line chemotherapy to surgery. CTRX, ceftriaxone; PIPC/TAZ, piperacillin/tazobactam; MEPM, meropenem.

**Figure 2:**
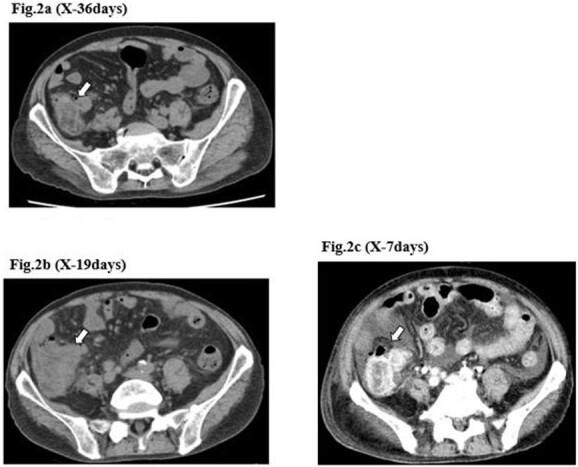
CT image of the ileocecal diverticulitis. The image shows that ileocecal diverticulitis (arrows) has occurred three times (**a–c**) until the diverticular perforation.

**Figure 3:**
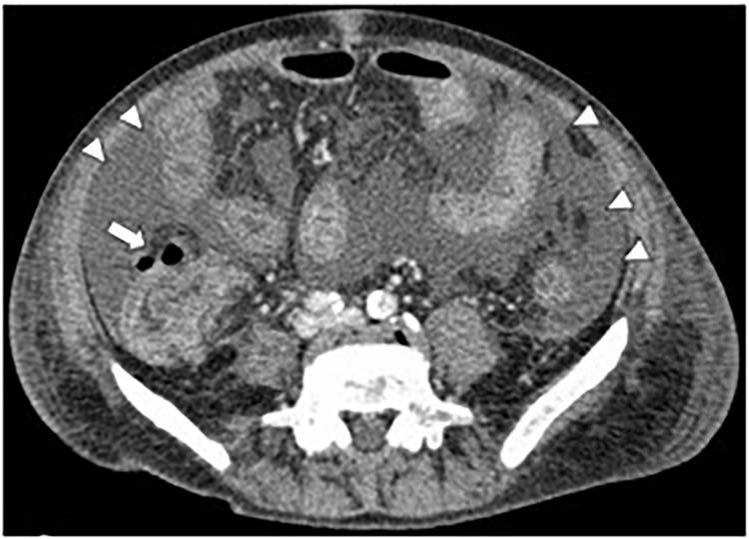
CT image of ascites (arrowheads) and free air (arrow).

**Figure 4:**
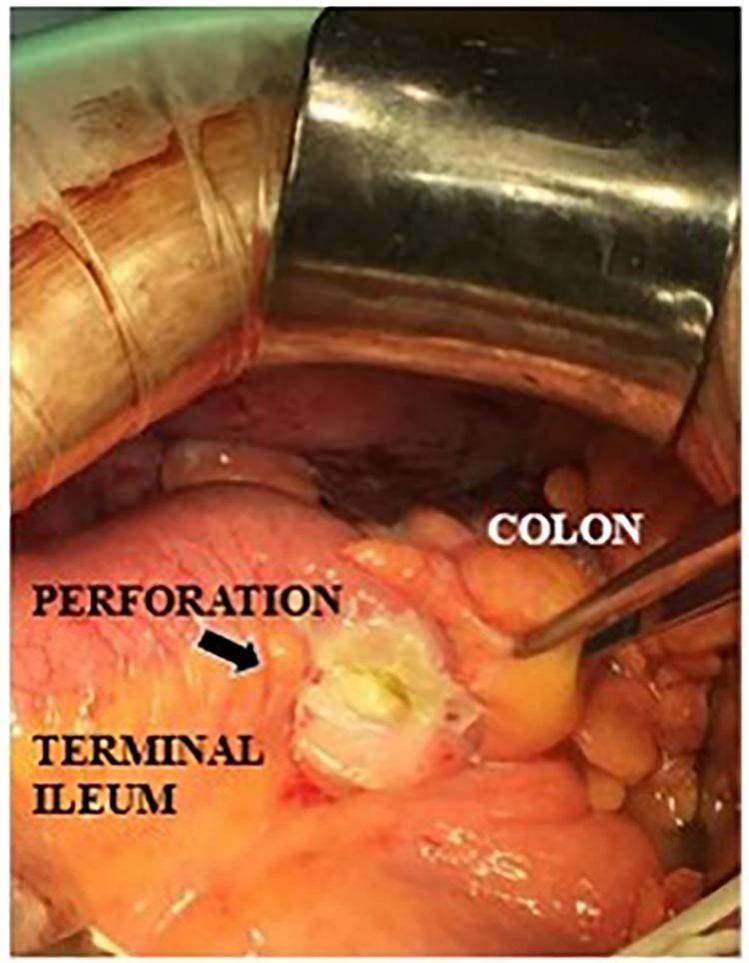
Intraoperative findings of a terminal ileal perforation on the mesenteric side (arrow).

A 71-year-old man was diagnosed with advanced non-small cell lung carcinoma and pleura and lymph node metastasis. He received carboplatin, paclitaxel, bevacizumab and atezolizumab every 3 weeks as first-line chemotherapy for 4 months. As the pleural dissemination worsened, docetaxel and ramucirumab were administered as second-line treatment. The patient had three episodes of ileocecal diverticulitis following chemotherapy, all of which were treated conservatively with antimicrobials and fasting therapy. The diverticulitis episodes and computed tomography (CT) images are shown in Figs 1 and 2a–c. During the fourth diverticulitis episode, the patient presented with fever and abdominal pain, worsening after 5 days. Physical examination revealed tenderness and rigidity throughout the abdomen. Vital signs were stable, but body temperature was 37.7°C. Laboratory data showed inflammation with a white blood cell (WBC) count of 21 400/μL and a C-reactive protein (CRP) level of 23.8 mg/dL. Abdominal CT imaging showed ascites and intra-abdominal free air around the right-side colon (Fig. 3). Hence, we suspected a right-side colon perforation with acute diffuse peritonitis and performed an emergency laparotomy with a midline incision. Surveying the small bowel revealed that ileal perforation occurred only a few centimeters proximal to the ileocolic valve (Fig. 4). The patient underwent ileocecal resection, ileostomy and a colonic mucous fistula.

**Figure 5:**
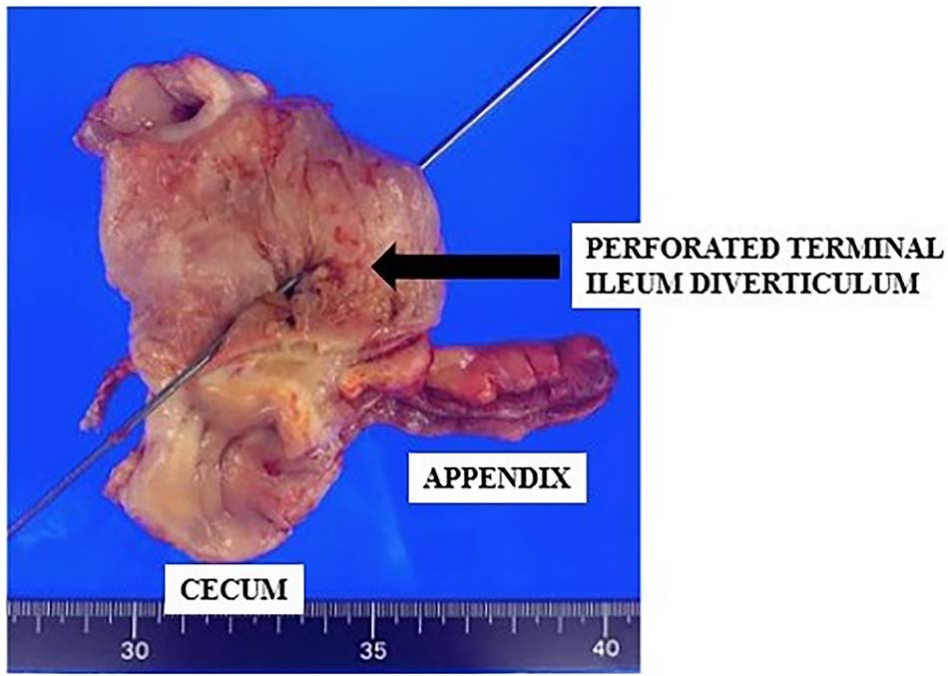
Resected specimen of ileocecum with the perforation of the terminal ileum (arrow).

**Figure 6:**
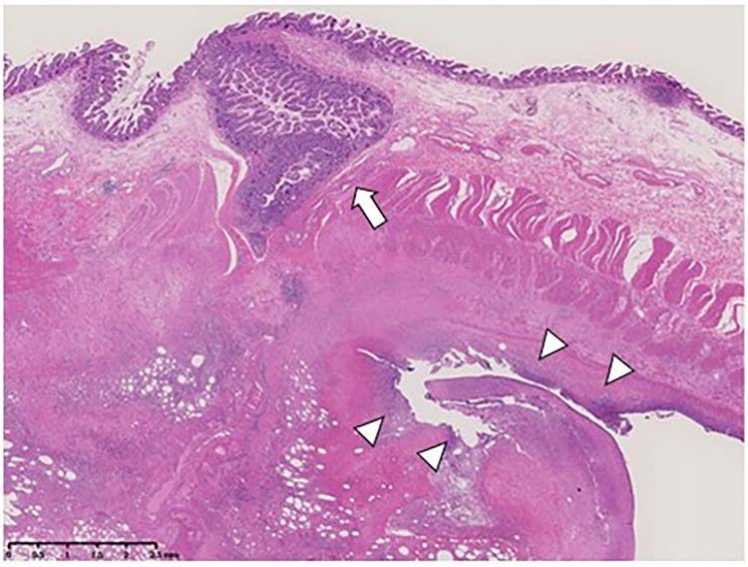
Specimen cross-section and microscopic examination (hematoxylin and eosin staining) of the terminal ileal diverticula (arrow) and perforation site (arrowheads).

The surgically resected specimen is shown in Fig. 5. Macroscopic findings indicated multiple ileal diverticula, with one perforated on the mesenteric border. Histopathological examinations revealed a pseudodiverticulum at the perforation where the mucosa was depressed through the muscularis propria. Additionally, inflammatory cell infiltration with abscess formation penetrating the serous surface was observed (Fig. 6).

The postoperative course was uneventful, and the patient was discharged 24 days after surgery. The patient did opt for chemotherapy again and died about 6 months after the surgery.

## DISCUSSION

Small bowel diverticulosis is rare, accounting for 0.02–7.1% of small bowel diverticulosis cases on imaging studies [4, 5] and 0.6–4.6% of autopsy cases [9, 10]. Reportedly, 80% of small intestinal diverticula are located in the jejunum, 15% in the ileum and 5% in both [10]. They are usually asymptomatic, but 30% are accompanied by chronic symptoms such as indigestion or irritable bowel syndrome-like symptoms, and only 10% are accompanied by inflammation, perforation, bleeding or obstruction [9, 10]. The small intestinal diverticulitis mortality rate is 21–45% because of low diagnostic rates and delayed treatment [10–12]. Diagnosing small intestinal diverticulitis may be difficult because of its rare occurrence. The small intestine’s location and free air may depend on body position and intestinal peristalsis, making it challenging to identify the perforation site and complicating imaging diagnosis, especially with extensive gut inflammation. Small intestine diverticula mostly occur on the mesenteric side because of the straight artery flowing into the tract, making it susceptible to diverticula.

Diverticula are more common in the jejunum than the ileum because of the large straight artery in the proximal jejunum, which promotes diverticula formation [9]. The patient had 2 months of repeated diverticulitis before the diverticula perforated, indicating weakened tissue from inflammation in the same area of the right colon to the ileum. Perforation and abscess risk may increase with each recurrence of colonic diverticulitis, but this is not documented in small bowel diverticulitis [13, 14]. No abscess or perforation was found in this case, and the patient was able to receive conservative care while continuing lung cancer treatment.

We conducted a MEDLINE search using the keywords ‘ileal diverticula’ and ‘perforation’ to identify previously reported cases of ileal diverticula perforation. Eight reports of diverticular penetration/perforation occurred exclusively in the ileum (Table 1) [4, 15–21], including one patient immunosuppressed by steroids. There was no chemotherapy-related case like the present case. Only one patient had a correct preoperative diagnosis. Seven of the eight patients underwent intestinal anastomosis, and only one underwent ileostomy, as in this case. Our patient was immunocompromised because of anticancer drug administration. As the patient had repeated diverticulitis episodes, the intestinal tract condition was weakened because of high inflammation, requiring creating an ileostomy to avoid complications related to the anastomosis.

**Table 1:** Literature review of ileal diverticular perforation (excluding Meckel’s diverticula and cases of penetration)

No	Reference	Year	Age/ sex	Past medical history	Medication	Preoperative diagnosis	Perforation site	Management	Cause of perforation
1	Miller *et al*. [16]	1970	49/M	NA	NA	Appendicitis	Mesenteric border	Ileocecal resection	NA
2	Ackerman [17]	1974	Appendectomy Inguinal hernia Heart failure	Various medication	Sigmoid diverticulitis	Mesenteric border	Right hemicolectomy	Diverticulitis	
3	Roses *et al*. [5]	1976	30/M	NA	NA	NA	Mesenteric border	Segmental ileal resection	Diverticulitis
4	Grana *et al*. [18]	2009	75/M	NA	NA	Penetration of terminal ileum	NA	Right hemicolectomy	Diverticulitis
5	Kothadia *et al*. [19]	2015	65/M	Hypertension Angina	NA	Sigmoid diverticulitis	NA	Elective surgery (Surgical technique was not described)	Diverticulitis
6	Thilakawardana *et al*. [20]	2017	29/M	Acute renal failure	Steroids	NA	Segmental ileal resection	Diverticulitis	
7	Ramzee *et al*. [21]	2020	69/M	NA	NA	Ileal diverticulitis	Mesenteric border	Segmental ileal resection Double-barrel ileostomy	NA
8	Saijo *et al*. [22]	2020	55/M	NA	NA	Penetration of terminal ileum	Mesenteric border	Ileocecal resection	Diverticulitis

M, male; F, female; NA, not available.

The risk of chemotherapy-induced gastrointestinal perforation reportedly increased with angiogenesis inhibitors—such as bevacizumab and ramucirumab—diverticulitis, peptic ulcer, radiation exposure, recent colonoscopy, primary tumor resection, bowel obstruction and multiple surgeries. It often occurred (within the first 6 months) following a few cycles of chemotherapy [22, 23]. Some case reports demonstrated that taxane-induced colitis can lead to colonic perforation requiring surgery. Paclitaxel may induce necrosis of the gastrointestinal mucosa by arresting mitosis of rapidly dividing colonic mucosal cells [24, 25]. In this case, the last dose of bevacizumab was administered more than 6 months before the small bowel perforation. The direct contribution of chemotherapy seems unlikely, and repeated diverticulitis from docetaxel or ramucirumab is suspected of having caused the perforation. The study’s limitations include the inherent limitations of case reports, such as the inability to generalize, prove causality and overinterpretation risk.

The perforation, in this case, may have been caused by repeated diverticulitis triggered by chemotherapy. Despite its low frequency, small bowel diverticula should be considered in patients experiencing unexplained fever and abdominal pain, as diverticulitis can occur in those undergoing chemotherapy. Preventive bowel resection is not required for diverticulitis during chemotherapy, but timely surgical intervention is crucial as diverticulitis in the small intestine has a high mortality rate.

## Data Availability

The data underlying this article will be shared on reasonable request to the corresponding author.
